# A Smartphone Camera Colorimetric Assay of Acetylcholinesterase and Butyrylcholinesterase Activity

**DOI:** 10.3390/s21051796

**Published:** 2021-03-05

**Authors:** Miroslav Pohanka, Jitka Zakova

**Affiliations:** Faculty of Military Health Sciences, University of Defense, Trebesska 1575, CZ-50001 Hradec Kralove, Czech Republic; jitka.zakova@unob.cz

**Keywords:** Carbamate, cholinesterase, diagnosis, Ellman’s assay, image analysis, inhibition, liver function test, organophosphate, point-of-care

## Abstract

Acetylcholinesterase (AChE) and butyrylcholinesterase (BChE) can serve as biochemical markers of various pathologies like liver disfunction and poisonings by nerve agents. Ellman’s assay is the standard spectrophotometric method to measure cholinesterase activity in clinical laboratories. The authors present a new colorimetric test to assess AChE and BChE activity in biological samples using chromogenic reagents, treated 3D-printed measuring pads and a smartphone camera as a signal detector. Multiwell pads treated with reagent substrates 2,6-dichlorophenolindophenyl acetate, indoxylacetate, ethoxyresorufin and methoxyresorufin were prepared and tested for AChE and BChE. In the experiments, 3D-printed pads containing indoxylacetate as a chromogenic substrate were optimal for analytical purposes. The best results were achieved using the red (R) channel, where the limit of detection was 4.05 µkat/mL for BChE and 4.38 µkat/mL for AChE using a 40 µL sample and a 60 min assay. The major advantage of this method is its overall simplicity, as samples are applied directly without any specific treatment or added reagents. The assay was also validated to the standard Ellman’s assay using human plasma samples. In conclusion, this smartphone camera-based colorimetric assay appears to have practical applicability and to be a suitable method for point-of-care testing because it does not require specific manipulation, additional education of staff or use of sophisticated analytical instruments.

## 1. Introduction

Two types of cholinesterases are known: acetylcholinesterase (AChE; EC 3.1.1.7) and butyrylcholinesterase (BChE; EC 3.1.1.8). BChE is an enzyme involved in the detoxification reaction of the first phase, as it can hydrolyze compounds like acetylsalicylic acid, cocaine, heroin and succinylcholine; additionally, because it is released from the liver to the blood, it can serve as a biochemical marker in a liver function test [1,2,3,4]. The second cholinesterase, AChE, is a physiologically substantial enzyme responsible for termination of cholinergic neurotransmission by hydrolysis of the neurotransmitter acetylcholine [5,6,7,8]. Both cholinesterases can be inhibited by various compounds. Some inhibitors are selective for either BChE or AChE. Highly toxic compounds like organophosphorus nerve agents (sarin, soman, tabun, VX) and former organophosphorus pesticides and drugs (paraoxon, malaoxon, metrifonate) are strong irreversible inhibitors of both AChE and BChE, while carbamate drugs and pesticides (carbofuran, pyridostigmine, rivastigmine, neostigmine) are pseudo-irreversible inhibitors of AChE and BChE [9,10,11]. Some current or former pesticides like parathion or malathion are not inhibitors of cholinesterases in vitro, but they can become active inhibitors like paraoxon or malaoxon by becoming metabolically activated. There is also a large group of cholinesterase inhibitors to which AChE is more sensitive than BChE because AChE can undergo cation–π interaction with the inhibitors due to its more developed peripheral anionic site and anionic gorge [12,13]; such inhibitors include caffeine, donepezil, huperzine, galantamine, aflatoxins and some heavy metal ions [14,15,16]. The measurement of cholinesterase activity has diagnostic significance. A decrease in AChE (using blood or tissue samples) can indicate poisoning by one of the aforementioned inhibitors. A decrease in BChE (using plasma or blood serum) is typically caused by a liver malfunction. When the activity of both AChE and BChE is reduced simultaneously, poisoning by an irreversible or pseudo-irreversible inhibitor is deemed to have occurred.

Ellman’s assay is commonly used to determine AChE and BChE activity. It is based on hydrolysis of acetylthiocholine (AChE assay) or butyrylthiocholine (BChE assay) into thiocholine and acetic acid, respectively, by a cholinesterase. Then, in the second step, thiocholine spontaneously reacts with (5,5-dithio-bis-(2-nitrobenzoic acid), providing yellow-colored anionic forms of 5-thio-2-nitrobenzoic acid and strongly absorbing visible light around 412 nm [17,18,19]. While there are other biochemical methods to determine cholinesterase activity, Ellman’s assay remains the standard and first choice in biochemical diagnoses. A modern alternative to Ellman’s assay is missing, especially a method suitable for a point-of-care testing. A simple colorimetric test to determine AChE and BChE activity is described as a potential alternative to Ellman’s assay. Digital photography was chosen as a measuring platform, making the method practical and suitable for point-of-care tests. Digital photography is popular due to its availability and simplicity, and may become a relevant tool in analytical chemistry. These advantages have already been recognized in the literature focused on various substrates [20,21,22,23,24,25].

## 2. Materials and Methods

### 2.1. Manufacturing of Measuring Pads and Smartphone Camera Holder

**Figure 1:**
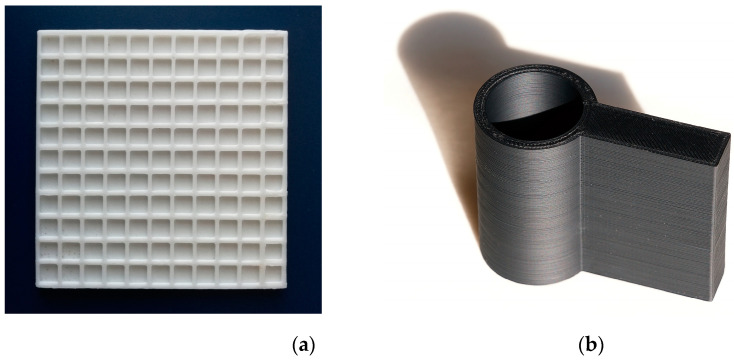
Photographs of a measuring pad (**a**) and smartphone camera holder (**b**) manufactured from acrylonitrile butadiene styrene (ABS) polymer.

Measuring pads and the camera holder for the colorimetric assay were manufactured using 3D printing technology. Black acrylonitrile butadiene styrene (ABS) was chosen for the holder and white ABS for the pad. They were manufactured using a Prusa i3 3D printer (Prusa Research; Prague, Czech Republic) using 2.9 mm ABS filaments (Prusa Research). The pads and camera holder were designed using the Autodesk 123D Design software (Autodesk; San Rafael, CA, USA) and the final proposals were processed in the Prusa3D Slic3r (Prusa Research). The measuring pads were 95 × 95 mm and contained 121 wells, each of which measured 6 × 6 mm and was 2 mm deep (internal volume 72 µL). The camera holder was 100 mm high with a 40 mm internal tube. Printing speed was 100 mm/s, layer depth was 0.2 mm, nozzle temperature was 285 °C and support desk temperature was 100 °C. A measuring pad and camera holder are depicted in Figure 1.

### 2.2. Measuring Pad Modification

The measuring pads were modified by chromogenic reagents. The following compounds were tested: 2,6-dichlorophenolindophenyl acetate, indoxylacetate, ethoxyresorufin and methoxyresorufin. The chromogenic reagents were dissolved in ethanol, up to concentrations of 100 mmol/L, and left to dry. Then, 10 µL of one of the aforementioned mixtures was added to one well and left to dry under laboratory conditions. Some wells were further treated by adding tetraisopropyl pyrophosphoramide (iso-OMPA). A mixture containing iso-OMPA 0.4 mmol/L in 10% w/w ethanol was prepared, and 10 µL of the mixture was added per well and left to dry under laboratory conditions. The modified measuring pads were stored in a dark and dry place until use. 

### 2.3. Camera-Based Colorimetric Assay of Cholinesterase Activity

Human AChE or BChE (Sigma–Aldrich; Saint Louis, MO, USA) or plasma from human volunteers was used in the experiments. A 40 µL sample was added to an individual well in the measuring pad and left to incubate for 60 min. The optimal incubation time was determined. The ABS measuring pads were placed into a wet chamber to prevent premature desiccation of the sample. After incubation, a camera holder with a smartphone (Huawei P10; Huawei Technologies, Shenzhen, Guangdong, China) was placed onto the pad, and 8-bit JPEG photographs were taken. The camera had activated LED light. Every photographed well was placed into the middle of camera view for accurate focusing, and each sample was photographed five times. 

### 2.4. Color Depth Determination

**Figure 2:**
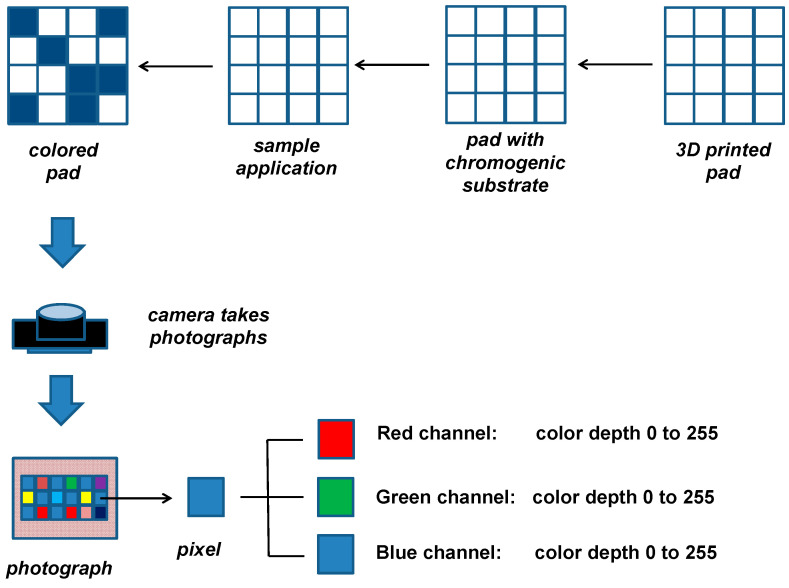
General principle of the colorimetric assay of cholinesterase activity.

Color depth was determined using the software GIMP 2.10.22 (free and open-source software). The photographs were analyzed in five randomly selected pixels 2 mm from the closest edges, and color depth was determined for red (R), green (G) and blue (B) channels. For 8-bit JPEG photographs, color depth has a value between 0 and 255 for each channel. The final change of color depth was expressed as an average value ± standard deviation. The general principle of the assay and the steps in processing are depicted in Figure 2.

### 2.5. Standard Assay of Cholinesterase Activity Determination

The activity of cholinesterases in the tested samples was determined by the standard spectral test in order to validate the results from the smartphone camera-based colorimetric assay. The standard spectral method was performed in disposable cuvettes with an optical length of 10 mm and total volume of 1.5 mL.

In the first step, 200 µL of 5,5′-dithiobis-2-nitrobenzoic acid 0.4 mg/mL was added into the cuvette and 40 µL of sample followed. Next, 560 µL of phosphate buffered saline pH 7.4 was added. In the case of the AChE assay in the presence of BChE, 100 µL of 1 mmol/L iso-OMPA was injected; in the other cases, 100 µL of saline was added to the cuvette. The reaction was started by the addition of acetylthiocholine chloride 1 mmol/L (AChE assay) or 100 µL butyrylthiocholine chloride (BChE assay). Absorbance was measured immediately and again after 5 min. Saline was used as a blank sample. Enzyme activity was calculated by considering yellow-colored anionic forms of 5-thio-2-nitrobenzoic acid and by using extinction coefficient ε = 14,150 l × mol^−1^ × cm^−1^.

## 3. Results and Discussion

Colorimetric substrates 2,6-dichlorophenolindophenyl acetate, indoxylacetate, ethoxyresorufin and methoxyresorufin were tested and compared mutually in the first part of the experiments. AChE and BChE were dissolved in the phosphate buffered saline, pH 7.4, with activity adjusted up 50 µkat/mL (activity expressed for substrates acetylthiocholine for AChE and butyrylthiocholine for BChE), and used as samples. The samples were applied on ABS polymer manufactured pads with the mentioned colorimetric substrates, and color depth change (60 min incubation) was measured in the R, G and B channels. Resulting data are depicted in Table 1. From the tested substrates, 2,6-dichlorophenolindophenyl acetate and indoxylacetate provided usable signal, but the changes in color depths for indoxylacetate were higher. Ethoxyresorufin and methoxyresorufin provide much lower signal and appear to be inappropriate for an assay of cholinesterase activity.

2,6-dichlorophenolindophenyl acetate and indoxylacetate were better substrates for BChE than AChE, and both substrates had higher changes in color depth in the R channel than in the G and B channels. In the case of 2,6-dichlorophenolindophenyl acetate and the R channel, the signal for AChE represented 61% of the signal for BChE. In the case of indoxylacetate and the R channel, the signal for AChE was equal to 57% of the signal for BChE. Considering the total signal, indoxylacetate provided a signal more than three times higher than that of 2,6-dichlorophenolindophenyl acetate. As a result, indoxylacetate was chosen as the chromogenic substrate for the assay of both AChE and BChE.

**Figure 3:**
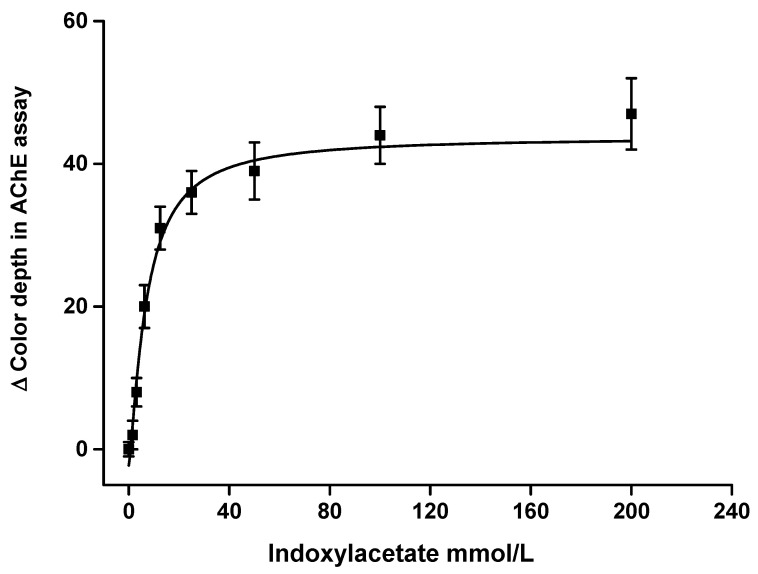
Saturation curve for indoxylacetate applied on a measuring pad with acetylcholinesterase (AChE) 50 µkat/mL. The concentration is expressed for 10 µL of sample. R (red) channel and 60 min incubation were chosen for this experiment. Standard error of the mean is expressed for five independent repeats.

**Figure 4:**
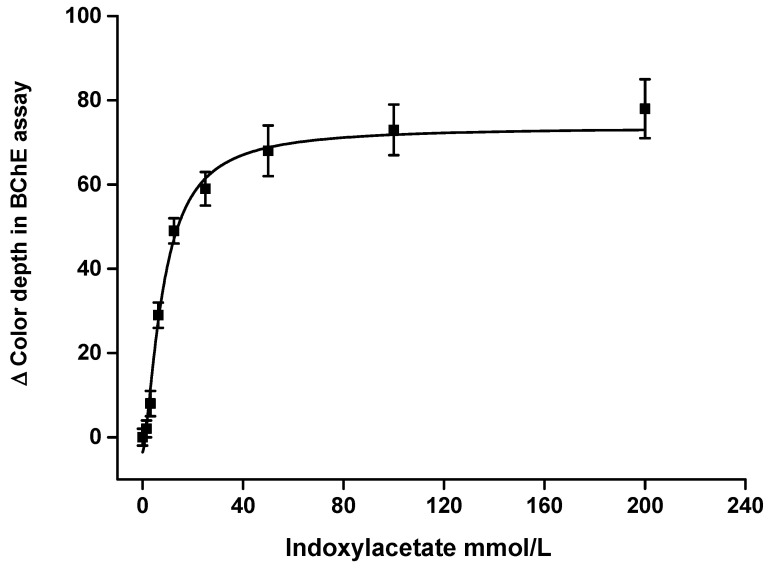
Saturation curve for indoxylacetate applied on a measuring pad with butyrylcholinesterase (BChE) 50 µkat/mL. The concentration is expressed for 10 µL of sample. R (red) channel and 60 min incubation were chosen for this experiment. Standard error of the mean is expressed for five independent repeats.

Substrate kinetics were measured using AChE and BChE as enzymes and indoxylacetate as a substrate. The saturation curves are depicted in Figure 3 for AChE and Figure 4 for BChE. The curves were fitted by the Michaelis–Menten equation. Maximal change of color depth for the tested samples was 44 for AChE and 74 for BChE. When using the measuring pad modification, the signal value was half of maximum when 7.0 mmol/L of indoxylacetate was used for the AChE assay and 8.1 mmol/L for the BChE assay. These values can be interpreted as Michaelis-like constants, but it should be reiterated that the concentration and volume were expressed for the indoxylacetate substrate using the measuring pad modification. The sample had four times higher volume (40 µL) than the volume of the applied indoxylacetate (10 µL). A decision was made to use a lower volume of reagents than that of the sample in order to ensure that the surface of the measuring well was covered more homogenously. The concentration of indoxylacetate chosen for the measuring pad modification was equal to 100 mmol/L. Saturation curve results plateaued using that level of concentration, and further increases of indoxylacetate had no significant beneficial effect. For this reason, a concentration of 100 mmol/L was considered optimal for the further experiments.

**Figure 5:**
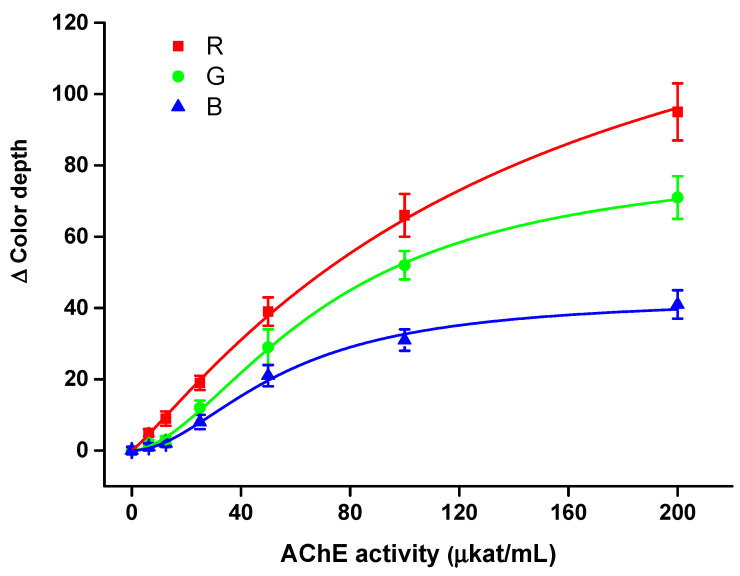
Calibration for acetylcholinesterase (AChE) using the smartphone camera-based colorimetric assay with the measuring pad modified with indoxylacetate. A 60 min incubation was chosen for this experiment. Standard error of the mean is expressed for five independent repeats. R red; G green; B blue.

**Figure 6:**
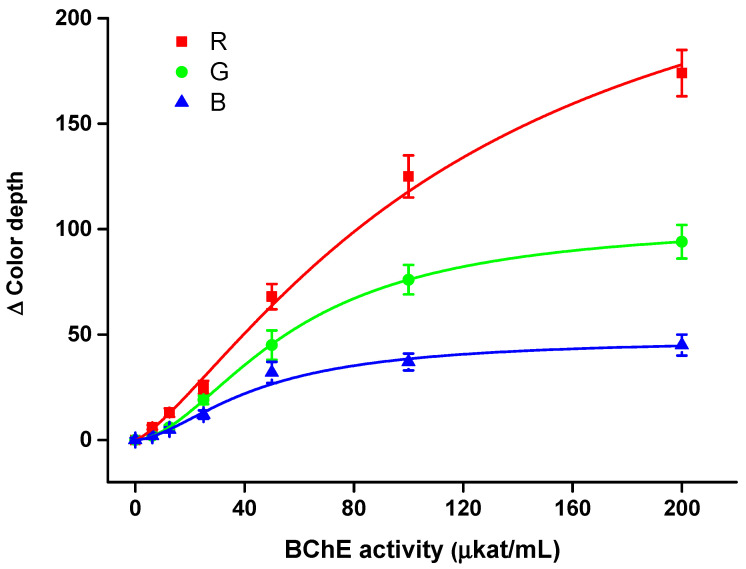
Calibration for butyrylcholinesterase (BChE) using the smartphone camera-based colorimetric assay with the measuring pad modified with indoxylacetate. A 60 min incubation was chosen for this experiment. Standard error of the mean is expressed for five independent repeats. R red; G green; B blue.

The smartphone camera-based colorimetric assay was properly calibrated for a pure solution of AChE and BChE in order to determine limits of detection and sensitivity to the analytes. The aforementioned optimal conditions (indoxylacetate injection 100 mmol/L with volume 10 µL and 60 min incubation) were used in this part of the experiment. All three optical channels were analyzed. Calibrations are depicted in Figure 5 for AChE and in Figure 6 for BChE. For both calibrations, R channel appeared to be the most sensitive, G channel less sensitive and B channel the least sensitive. Limits of detection, calculated as triplicate of signal to blank sample (S/N = 3), were determined for every channel. 

The AChE assay had a detection limit of 4.38 µkat/mL, 11.0 µkat/mL and 14.3 µkat/mL for R channel, G channel and B channel, respectively, and a coefficient of determination (r^2^) for calibration of 0.998, 0.995 and 0.994, respectively. Relative standard deviation was equal to 10% for R channel, 16% for G channel and 14% for B channel when a sample containing AChE 50 µkat/mL was analyzed.

The BChE assay was more sensitive compared with the AChE assay. The best results were obtained using the R channel, where the limit of detection was 4.05 µkat/mL and r^2^ = 0.989; the G channel’s results were 8.16 µkat/mL and r^2^ = 0.999, and the B channel’s were 9.10 µkat/mL and r^2^ = 0.987. The relative standard deviation was equal to 9% for the R channel, 15% for the G channel and 16% for the B channel when a sample containing BChE 50 µkat/mL was analyzed.

Considering the aforementioned results, all the color channels could be used for the assay, but the R channel appeared to be optimal, and was thus chosen as the first choice for further measurements. The better limits of detection for BChE than AChE were not surprising when one considers the higher affinity of BChE for indoxylacetate, as mentioned. 

**Figure 7:**
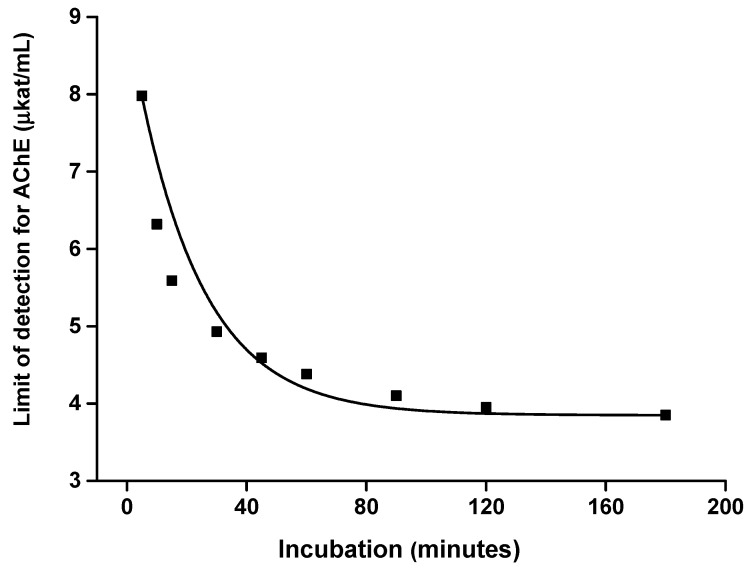
Effect of incubation time on the limit of detection when acetylcholinesterase (AChE) was measured by a camera-based colorimetric assay and the measuring pad was modified with indoxylacetate. R (red) channel was used for the assay. Standard error of the mean is expressed for five independent repeats.

**Figure 8:**
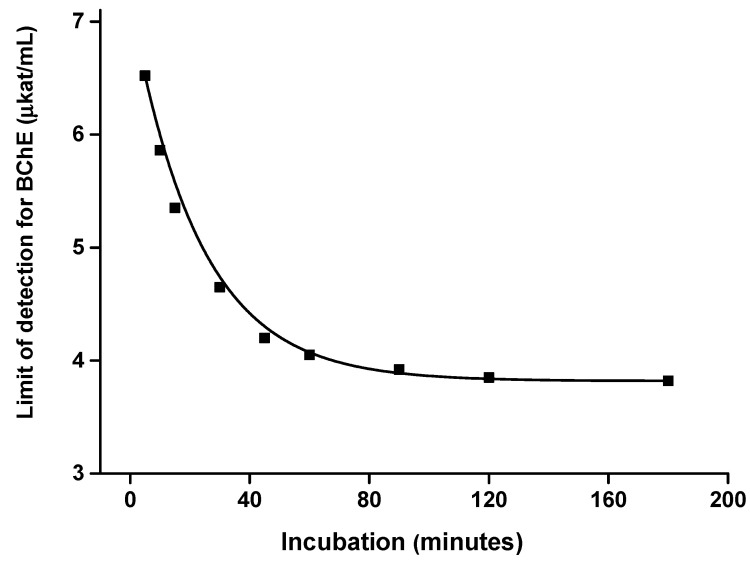
Effect of incubation time on the limit of detection when butyrylcholinesterase (BChE) was measured by a camera-based colorimetric assay and the measuring pad was modified with indoxylacetate. R (red) channel was used for the assay. Standard error of the mean is expressed for five independent repeats.

**Figure 9:**
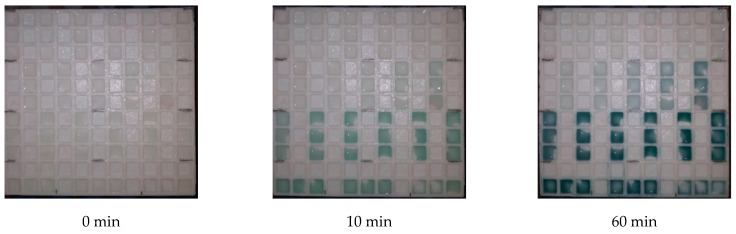
Illustrative photographs showing 42 of the total 121 wells with solutions of 40 µL butyrylcholinesterase (BChE) (0–100 µkat/mL). The measuring pad was photographed in the beginning of experiment, after 10 min and after 60 min.

The effect of incubation time on the limit of detection was investigated as well. Calibrations for AChE (Figure 7) and BChE (Figure 8) were done at various incubation times and the limits of detection were expressed against the incubation. Considering these results, an incubation time of up to 40 min has significant effects on the received limit of detection. The plateau of the effect is reached at an incubation time of 60 min. Thus, incubation time of 60 min was chosen as optimal. Illustrative photographs from the experiment are depicted in Figure 9.

**Figure 10:**
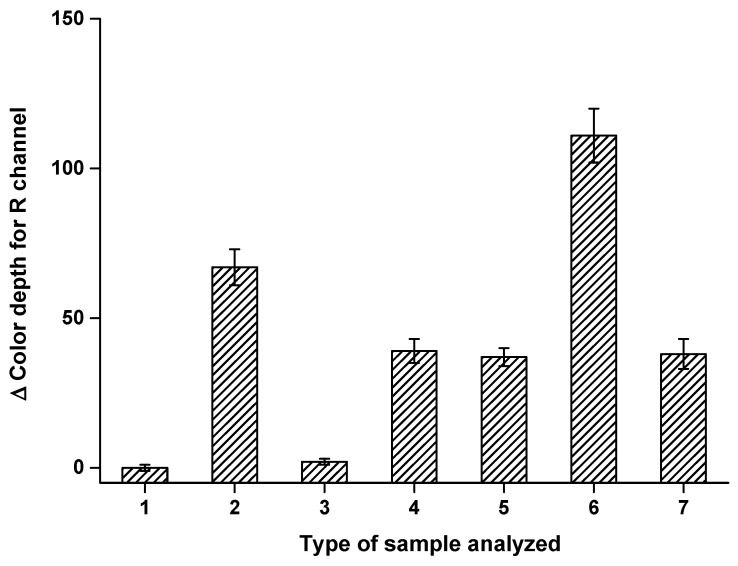
The effect of tetraisopropyl pyrophosphoramide (iso-OMPA) on the smartphone camera-based colorimetric assay and the measuring pad modified with indoxylacetate assay of acetylcholinesterase (AChE), butyrylcholinesterase (BChE) and a mixture of AChE and BChE. Type of sample analyzed: (**1**) application of saline solution to the well with indoxylacetate and iso-OMPA; (**2**) 40 µL AChE 50 µkat/mL; (**3**) 40 µL AChE 50 µkat/mL and 0.1 mmol/mL iso-OMPA; (**4**) 40 µL BChE 50 µkat/mL; (**5**) 40 µL BChE 50 µkat/mL and 0.1 mmol/mL iso-OMPA; (**6**) 40 µL 50 µkat/mL BChE and 50 µkat/mL BChE; (**7**) 40 µL 50 µkat/mL AChE, 50 µkat/mL BChE and 0.1 mmol/L iso-OMPA. Standard error of the mean is expressed for five independent repeats. R red.

The effect of iso-OMPA on the AChE assay was tested; it was used as a reagent to determine AChE activity in the presence of BChE, as it is a selective inhibitor of BChE but not of AChE [26,27,28]. The following samples were tested in the wells treated with indoxyacetate: (1) saline solution and iso-OMPA; (2) 40 µL BChE 50 µkat/mL; (3) 40 µL BChE 50 µkat/mL and 0.1 mmol/L iso-OMPA; (4) 40 µL AChE 50 µkat/mL; (5) 40 µL AChE 50 µkat/mL and 0.1 mmol/mL iso-OMPA; (6) 40 µL 50 µkat/L AChE and 50 µkat/mL BChE; (7) 40 µL 50 µkat/mL AChE, 50 µkat/mL BChE and 0.1 mmol/L iso-OMPA. Results from this test are depicted in Figure 10. The effect of iso-OMPA was evaluated using analysis of variance (ANOVA) for P = 0.05 and P = 0.01. The iso-OMPA had no significant effect on the AChE assay. The BChE assay is not possible in the presence of iso-OMPA because the signal significantly dropped due to iso-OMPA (P = 0.01) and became insignificant when only saline was applied. When a mixture of AChE and BChE was analyzed, the iso-OMPA was able to fully inhibit BChE, resulting in a decrease in the signal to the same level (no significant difference) as that caused by the same activity of pure AChE. The experiment shows that the measuring pads treated with iso-OMPA can be used whenever activity of only AChE should be assayed, or that two measurements (with iso-OMPA and without iso-OMPA) can be performed, with the difference between the two signals interpreted as being caused by BChE only.

Interferences in the assay were tested and variants of the interference causing false negatives of a cholinesterase assay and false positivity were considered. Human serum albumin 10 mg/mL, 10% ethanol, human hemoglobin 10 mg/mL, glucose 10% w/w, ascorbic acid 10 mg/mL and acetylsalicylic acid 10 mg/mL were tested individually or in a mixture in cases where the aforementioned concentrations (final concentration in the sample) were below 50 µkat/mL BChE or 50 µkat/mL AChE (final activities in the sample). Saline served as a negative control assay. Solutions containing 50 µkat/mL BChE or 50 µkat/mL AChE without the tested substances served as a positive control. The tested substances caused no significant signal (no significant difference) compared with results from the saline assay. Assays of AChE and BChE in the mixture with the tested substances were not significantly different from assays of pure AChE or BChE. The assay appears to be resistant to interference by commonly occurring sample compounds.

**Figure 11:**
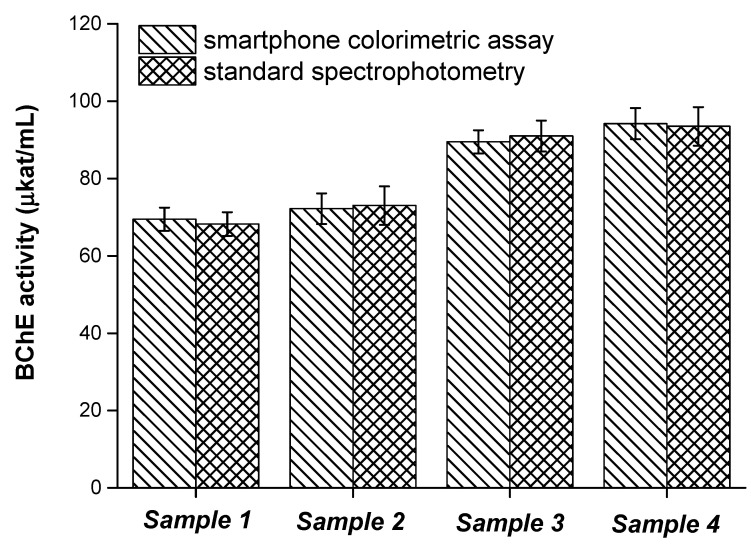
The assay of plasma samples by standard spectrophotometry and by the newly developed smartphone camera-based colorimetric assay. A 60 min incubation was chosen for this experiment. Standard error of the mean is expressed for five independent repeats. BChE butyrylcholinesterase.

The smartphone camera-based colorimetric test was compared with the standard spectrophotometric assay of cholinesterase activity. The aforementioned calibration served to assess activity calculation by using the changes in color depth. Four plasma samples were tested for BChE activity. Results from this experiment are depicted in Figure 11. No significant difference by ANOVA was recorded on the tested probability levels P = 0.05 and P = 0.01 when the same sample was analyzed by the two methods. The validation of our smartphone camera-based colorimetric test confirms that it can be considered a reasonable method for cholinesterase activity determination and that its results are similar to those obtained by using a standard spectrophotometric assay. The overall simplicity and applicability of the smartphone camera-based colorimetric test in point-of-care conditions is the major advantage of this newly developed assay.

## 4. Conclusions

A smartphone camera-based colorimetric assay of cholinesterase activity proved to be a reliable tool for a point-of-care test where AChE, BChE or both play a role as a biochemical marker. The assay provides results similar to those of standard spectrophotometry. Compared to standard spectrophotometry, the smartphone camera-based colorimetric assay needs only one additional step (represented by a sample application); no other manipulation with the sample or specific treatment of the sample is necessary. Specifications of the assay are promising for a point-of-care test and make it easily adaptable in the current clinical and home care praxis. The smartphone camera-based colorimetric assay also meets the common requirements and expectations of point-of-care tests: simplicity, portability, no elaborative manipulations with samples, low cost, no use of harmful reagents, etc. [29,30,31,32,33,34,35]. Additionally, it meets the requirements of thin layer detectors like paper biosensors [36].

## Figures and Tables

**Table 1:** Changes of color depths for acetylcholinesterase (AChE) and butyrylcholinesterase (BChE) assay ^1^.

Substrate	Change of Color Depth for BChE Assay	Change of Color Depth for AChE Assay
2,6-dichlorophenolindophenyl acetate	R = 18 ± 3; G = 12 ± 5; B = 14 ± 2	R = 11 ± 5; G = 5 ± 4; B = 4 ± 2
indoxylacetate	R = 68 ± 6; G = 45 ± 7; B = 32 ± 5	R = 39 ± 4; G = 29 ± 5; B = 21 ± 3
ethoxyresorufin	R = 2 ± 1; G = 1 ± 0; B = 3 ± 2	R = 9 ± 4; G = 8 ± 2; B = 4 ± 3
methoxyresorufin	R = 3 ± 2; G = 2 ± 1; B = 1 ± 1	R = 5 ± 3; G = 4 ± 2; B = 5 ± 3

^1^ AChE and BChE 50 µkat/mL assay using various colorimetric substrates applied in 10 µL of 100 mmol/L on the measuring pad and left to incubate for 60 min. Standard error of the mean is expressed for five independent repeats. R red; G green; B blue.

## Data Availability

All data are provided in this work.
